# Trajectory of Estimated Glomerular Filtration Rate and Malnourishment Predict Mortality and Kidney Failure in Older Adults With Chronic Kidney Disease

**DOI:** 10.3389/fmed.2021.760391

**Published:** 2021-11-29

**Authors:** Shuo-Chun Weng, Chyong-Mei Chen, Yu-Chi Chen, Ming-Ju Wu, Der-Cherng Tarng

**Affiliations:** ^1^College of Medicine, National Chung Hsing University, Taichung, Taiwan; ^2^Center for Geriatrics and Gerontology, Division of Nephrology, Department of Internal Medicine, Taichung Veterans General Hospital, Taichung, Taiwan; ^3^Institute of Clinical Medicine, School of Medicine, College of Medicine, National Yang Ming Chiao Tung University, Taipei, Taiwan; ^4^Institute of Public Health, College of Medicine, National Yang Ming Chiao Tung University, Taipei, Taiwan; ^5^Institute of Clinical Nursing, College of Nursing, National Yang Ming Chiao Tung University, Taipei, Taiwan; ^6^School of Medicine, Chung Shan Medical University, Taichung, Taiwan; ^7^Rong Hsing Research Center for Translational Medicine, Institute of Biomedical Science, College of Life Science, National Chung Hsing University, Taichung, Taiwan; ^8^Graduate Institute of Clinical Medical Science, School of Medicine, China Medical University, Taichung, Taiwan; ^9^Department and Institute of Physiology, National Yang Ming Chiao Tung University, Taipei, Taiwan; ^10^Division of Nephrology, Department of Medicine, Taipei Veterans General Hospital, Taipei, Taiwan; ^11^Center for Intelligent Drug Systems and Smart Bio-devices (IDS2B), National Yang Ming Chiao Tung University, Hsinchu, Taiwan

**Keywords:** eGFR trajectory, kidney failure, malnourishment, mortality, older adults

## Abstract

**Objective:** The trajectory patterns of estimated glomerular filtration rates (eGFR) in chronic kidney disease (CKD) older adults with malnourishment and their association with subsequent patient outcomes have not been elucidated. We aimed to assess the eGFR trajectory patterns for predicting patient survival and kidney failure in the elderly without or with malnourishment.

**Materials and Methods:** Based on a prospective longitudinal cohort, CKD patients aged 65 years or older were enrolled from 2001 to 2013. Among the 3,948 patients whose eGFR trajectory patterns were analyzed, 1,872 patients were stratified by the absence or presence of malnourishment, and 765 patients were identified and categorized as having malnourishment. Four eGFR trajectory patterns [gradual decline (T0), early non-decline and then persistent decline (T1), persistent increase (T2), and low baseline and then progressive increase (T3)] were classified by utilizing a linear mixed-effect model with a quadratic term in time. The malnourishment was defined as body mass index < 22 kg/m^2^, serum albumin < 3.0 mg/dL, or Geriatric Nutritional Risk Index (GNRI) < 98. This study assessed the effectiveness of eGFR trajectory patterns in a median follow-up of 2.27 years for predicting all-cause mortality and kidney failure.

**Results:** The mean age was 76.9 ± 6.7 years, and a total of 82 (10.7%) patients with malnourishment and 57 (5.1%) patients without malnourishment died at the end of the study. Compared with the reference trajectory T0, the overall mortality of T1 was markedly reduced [adjusted hazard ratio (aHR) = 0.52, 95% confidence interval (CI) 0.32–0.83]. In patients with trajectory, T3 was associated with a high risk for kidney failure (aHR = 5.68, 95% CI 3.12–10.4) compared with the reference, especially higher risk in the presence of malnourishment. Patients with high GNRI values were significantly associated with a lower risk of death and kidney failure, but patients with malnourishment and concomitant alcohol consumption had a higher risk of kidney failure.

**Conclusions:** Low baseline eGFR and progressively increasing eGFR trajectory were high risks for kidney failure in CKD patients. These findings may be attributed to multimorbidity, malnourishment, and decompensation of renal function.

## Introduction

Longitudinal eGFR change in patients with CKD is often nonlinear, and most people with CKD have major clinical events and die before reaching kidney failure. In previous observational studies, patients with CKD experienced the possibility of non-progression of renal function, stable kidney disease with accelerated deterioration, and relentlessly fast eGFR decline into kidney failure (1–3). However, a paradoxical association exists between high eGFR and fast eGFR decline, which were found to be correlated with increased risk of death, but were associated with a comparatively lower risk of developing kidney disease (2, 4). For the above reasons, other parameters, such as serum creatinine, eGFR value, nutritional assessment, and severity of comorbid conditions are repeatedly measured to predict patients' outcomes. Traditional methods, such as baseline eGFR by category, absolute change in eGFR value, annual eGFR decline or annual percentage change of eGFR, the velocity of eGFR slopes, and eGFR variability, have been widely used for predicting cognitive deterioration, cardiovascular risk, renal outcome, and patient mortality (5–11). However, a more comprehensive picture of disease progression can be obtained using the eGFR trajectory pattern classification, which has been proven superior to baseline eGFR since it can capture the whole process of eGFR measurements and can take non-linear patterns into account (12), whereas baseline eGFR at cohort entry or longitudinal eGFR values showing linear trends provide more limited information.

Malnourishment, which was caused by CKD, respiratory disease, cancer, and cardiovascular disease, was reported to possibly contribute to high eGFR mortality, especially in older adults (4, 13). The main reasons for developing malnourishment were comorbid illnesses, oxidative and carbonyl stress, nutrient loss through diseases, anorexia and low nutrient intake, uremic toxins, and a decreased clearance of inflammatory cytokines (14). More importantly, older adults have age-related changes in appetite, swallowing, and dental problems, and those issues are predisposed to decreased food intake and malnourishment, especially inadequate fluid or protein intake. Thus, age-related multimorbidity and loss of muscle mass would tend to contribute to a progressively increasing eGFR trajectory and make an assessment of long-term clinical outcomes based on short-term measurements of renal function more difficult (15, 16). Besides, the U-shaped relationship between low body mass index (BMI) and sarcopenic obesity has been strongly associated with all-cause and cardiovascular mortality in older adults (17, 18), and our previous study revealed that the increased risk of mortality in subjects with high eGFR was primarily attributable to the presence of malnutrition-inflammation-cachexia syndrome (4). However, few studies have discussed the relationships among eGFR trajectories, BMI, and nutritional status and their effects on clinical outcomes.

Clinically, great diversity in longitudinal eGFR measurements exists. These patterns reveal different clinical pictures of kidney function. Taking these characteristics of longitudinal eGFR profiles into account would provide more information on the condition of kidneys compared with using baseline eGFR or decline rate. Therefore, we conducted a prospective longitudinal cohort study using the eGFR trajectory to predict patient survival and renal outcome in CKD older adults without or with malnourishment.

## Methods

### Patients and Data Sources

**Figure 1:**
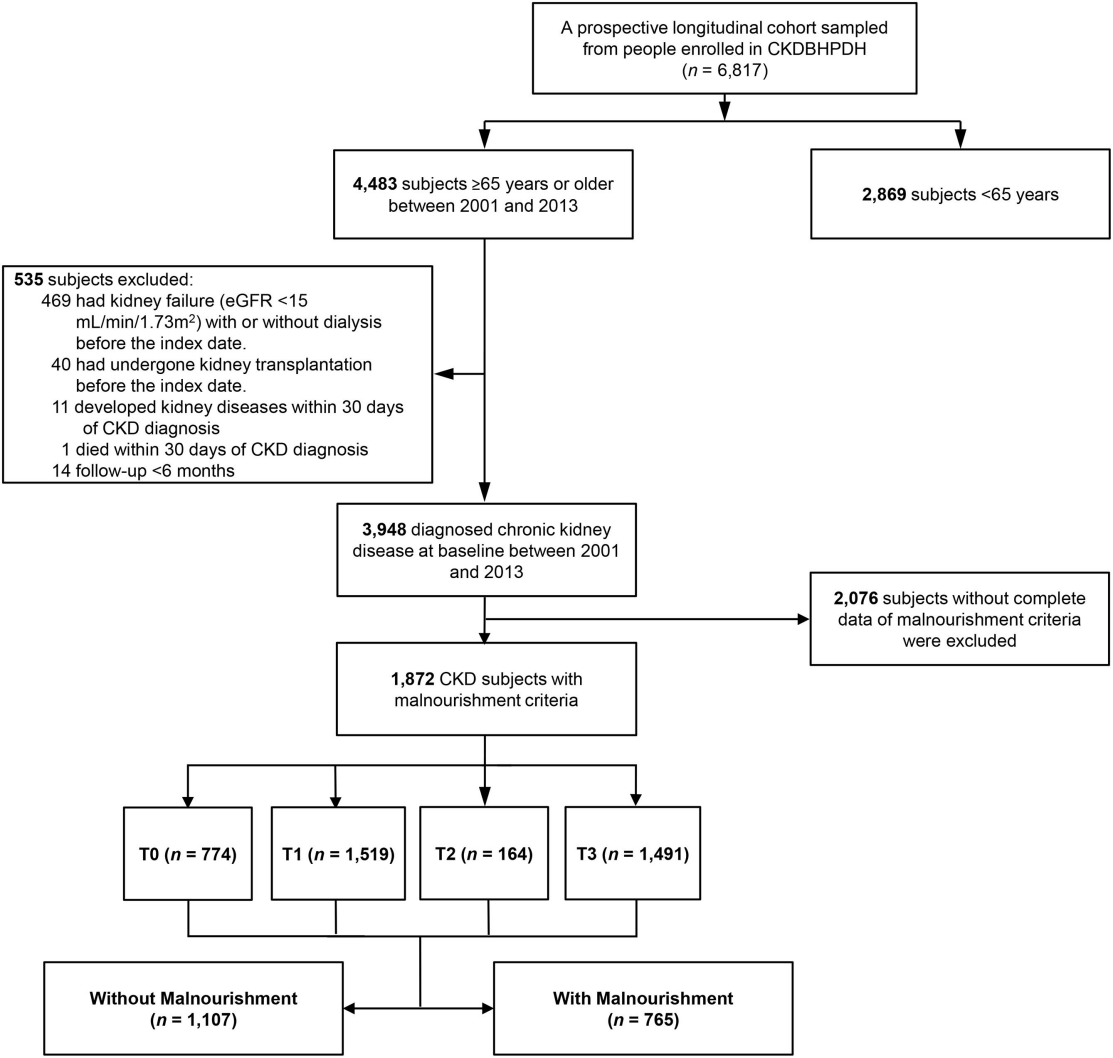
Flow chart of subject selection for the study cohort. A total of 3,948 older adults with a history of chronic kidney diseases (CKD) was identified in the Chronic Kidney Disease division of the Bureau of Health Promotion, Ministry of Health and Welfare (CKDBHPDH) from 2001 to 2013. Among the 3,948 patients, 2,076 patients without complete data of malnourishment criteria were excluded. 1,872 participants whose eGFR trajectory patterns were analyzed and were stratified by the absence or presence of malnourishment, and 765 patients were identified and categorized as having malnourishment. T0–T3: eGFR trajectory patterns.

Data from the CKD cohort were obtained from patients' medical records at Taichung Veterans General Hospital (TCVGH_ CKD cohort, Figure 1). All registration data were obtained from the Chronic Kidney Disease division of the Bureau of Health Promotion, Ministry of Health and Welfare (CKDBHPDH) (19). CKD was defined as eGFR < 60 ml/min more than 3 months, urine albumin/creatinine ratio > 30 mg/g (20), urine protein/creatinine ratio > 0.2 mg/g (21) or abnormal kidney image. In the TCVGH_CKD cohort, we identified men and women older than 65 years of age with CKD (*n* = 3,948) from five counties and cities in central Taiwan from December 1, 2001, to July 31, 2013. Among the 3,948 patients, 2,076 patients without complete data of malnourishment criteria were excluded. 1,872 participants whose eGFR trajectory patterns were analyzed and were stratified by the absence or presence of malnourishment, and 765 patients were identified and categorized as having malnourishment. The patients in the TCVGH_CKD cohort had at least 3 outpatient eGFR records per year, based on data obtained from the Taiwan Society of Nephrology (TSN). However, before January 01, 2014, serum creatinine was measured by the Jaffe method using a Beckman Synchron CX5 analyzer (USA) calibrated under the standards of the Chinese National Laboratory Accreditation program. After January 01, 2014, the enzymatic method (CYGNUS AUTO CRE) and spectrophotometry were used. Patients were excluded if they had undergone kidney transplantation or had irreversible kidney failure with or without dialysis before the index date. The TCVGH_CKD database included patients with early CKD (CKD stage 1, 2, 3A) and pre-kidney failure (CKD stage 3B, 4, 5), and all participants were followed up for longer than 6 months, until February 07, 2014, to prevent lead-time bias.

### Patient and Public Involvement Statement

Older adults with CKD are often undernourished and physically inactive, which contributes to sarcopenia and frailty. The trajectory of eGFR is a potential surrogate marker to demonstrate the causal relationship among muscle mass, aging changes in the kidneys, renal survival, and patient mortality. Outcome measurements and study design were approved by the institutional review board of TCVGH informed by patients' priorities, experience, and preferences according to the Declaration of Helsinki. Using administration data from the CKDBHPDH, a prospective cohort was selected and enrolled. All subjects gave their informed consent for inclusion before they participated in the study. Although informed consent was required, multidisciplinary and geriatric care did not interfere with clinical decisions related to patient care. Whether written informed consent was given by participants for their clinical records to be used in this study, every consent was obtained before the patient records/information was anonymized and de-identified before analysis. The results will be disseminated to study participants if the dissertation would be accepted. The study protocol was affirmed by the Ethics Committee of Taichung Veterans General Hospital and Taipei City Hospital.

### Definition of eGFR Trajectory Patterns

**Figure 2:**
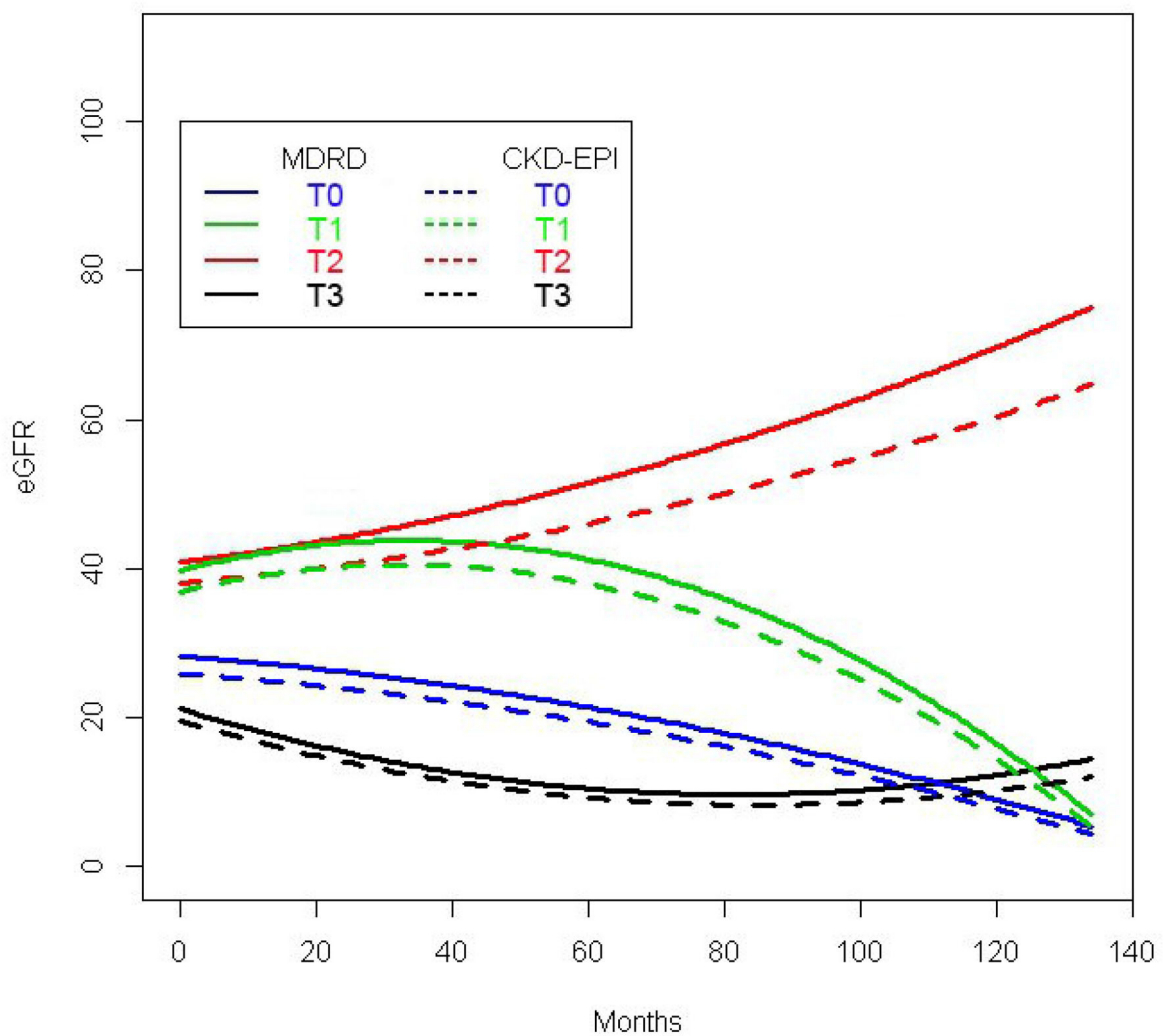
Four eGFR trajectories in the CKD cohort. Pattern 1 (T0): solid blue line, gradual eGFR decline; pattern 2 (T1): solid green line, early non-decline and then persistent decline; pattern 3 (T2): solid red line, persistently increasing eGFR; pattern 4 (T3): solid black line, low baseline eGFR with the early decline and then progressively increasing eGFR. Four eGFR trajectories with the CKD-EPI equations were also shown (dotted blue, green, red, and black line).

Each patient was classified into one of four groups based on the four-variable Modification of Diet in Renal Disease (MDRD) Study equation (22, 23). Those eGFR data were obtained prospectively, and the number of eGFR measurements is various with ≥ 3 before enrollment. To stratify the eGFR trajectory patterns, we first applied a linear mixed-effects model with a quadratic term in time for repeatedly measured eGFR values (polygonal lines in Supplementary Figure 1) (24). More specifically, the eGFR of each patient is modeled as a quadratic function of time to examination since treatment with all terms having random effects. In addition to the estimation of the fixed effects, we also obtained the empirical Bayes estimates of each patient's random effects, which are distributed random variables with a mean zero. By plugging these estimates, a patient's trajectory is fitted by the polynomial of degree 2 with the corresponding estimated random effects and fixed effects as coefficients. Based on that, we can classify an individual into one of the 4 eGFR trajectories. eGFR trajectory pattern 1 (T0), which is characterized by the baseline eGFR 27.9 ± 9.8 and a median 9.25-year later the eGFR estimated as 11.3 ± 10.3 ml/min/1.73m^2^ per year (25), was defined to be stably slow progression accounting for 19.6% of patients (Figure 2). Thirty-five empirical trajectories of eGFR_MDRD randomly selected from each group are drawn in the following figure (polygonal lines in Supplementary Figure 1) combining with the classified eGFR_MDRD trajectories for these 4 groups. Each smooth curve is the average of the observed trajectories in the group corresponding to the same color. It can be seen that the estimated and smooth trajectories fitted all patients' empirical eGFR trajectories well. Trajectory pattern 2 (T1), which is characterized by early non-decline (< -0.43 to −0.48 ml/min/1.73 m^2^ per year) (2) and then persistent decline of more than −3 ml/min/1.73 m^2^ per year, accounted for 38.4% of patients. The turning point of the eGFR trajectory was determined by the joint model using latent random effects (26). Trajectory pattern 3 (T2), which is characterized by persistently increasing eGFR without any decreases, accounted for 4.2% of patients. Trajectory pattern 4 (T3), which is characterized by an initial low eGFR of 20 ml/min/1.73 m^2^ and mild progression (≥ -2 ml/min/1.73 m^2^ per year for every month of the period or ≥ -1 ml/min/1.73 m^2^ per year, which corresponded roughly to the average age-related decline in GFR) (1) in the first 5 years, followed by progressive increasing eGFR (T3) values without any decreases, accounted for 37.8% of patients. Sensitive analyses with the CKD-EPI equations (27) were also shown in Figure 2.

### Assessment of Malnourishment

The clinical presence of malnourishment in older adults was determined based on the following indicators: body mass index < 22 kg/m^2^, serum albumin < 3.0 g/dL, or GNRI < 98 (4). The GNRI formula is as follows: GNRI = (1.489 × albumin in gram per liter) + (41.7 × present/ideal body weight). The ideal body weight was calculated according to the Lorentz formula, which takes into account a patient's height and sex: for men, height – 100 – ([height – 150]/4); for women, height – 100 – ([height – 150]/2.5) (4, 28–30).

### Availability of Data and Imputation

There are missing values for various variables in the judgment of malnourishment in 3,948 patients, and we listed the detailed numbers of patients with abnormal levels of albumin, BMI, or GNRI in Supplementary Table 1. All missing values were imputed using multiple imputations with the FCS (Fully Conditional Specification) imputation method (31). Ten imputations were performed. All the estimates and the corresponding standard error estimates in each imputation were properly combined.

### Covariates

Covariates included age, gender, smoking, alcohol, initial eGFR, diabetes mellitus (DM), hypertension (HTN), cardiovascular diseases, malignancy, malnourishment components (4, 28), the classified eGFR trajectory patterns, serum uric acid, HbA1c, and medication. Baseline comorbidities were ascertained at the time of study entry. Comorbid conditions were self-reported by the patients or retrieved from electronic records. The presence of cardiovascular diseases, i.e., coronary artery disease, stroke, congestive heart failure, arrhythmia, and peripheral arterial disease, was determined using patients' medical records.

### Study Outcomes

Mortality was obtained from the death registry system and coded from death certificates according to the International Classification of Diseases (ICD)-9 or ICD-10. The primary outcome was all-cause mortality, and the secondary outcome was kidney failure. Patients were identified as having kidney failure if they had undergone maintenance dialysis or short-term dialysis before death.

### Statistical Analyses

Based on the empirical Bayes estimates of all random effects, each patient's eGFR profile was estimated. The results revealed four patterns of eGFR trajectories. Each patient was classified into one of the four trajectory patterns according to his/her estimated eGFR profile. To construct eGFR trajectories, the longitudinal eGFR pattern was followed for nearly 140 months (Figure 2). The baseline demographics are presented as number and percentage for categorical variables and as mean with standard deviation (SD) or median with interquartile range (IQR) for continuous variables. We compared the characteristics of patients with and without malnourishment and among the different eGFR trajectory patterns using a Chi-square test for categorical variables and one-way ANOVA for continuous variables. The *post-hoc* analysis was conducted to compare all pairs of subgroups. These calculations were conducted using SAS PROC MI and PROC MIANALYZE. Multinomial logistic regression analysis was applied to evaluate the associations between eGFR trajectory patterns and factors, including demographic variables, comorbid conditions, lifestyle, and nutritional status of older people. The primary outcome, all-cause mortality, and the secondary outcome, kidney failure, were analyzed using cause-specific hazard models for competing risk data. All variables, including the classification of eGFR trajectory patterns, were adjusted in the survival analysis. In all tests, a *p* < 0.05 was considered statistically significant. Statistical analyses were implemented using R statistical software, version 3.4.2, and SAS, version 9.4 (SAS Institute Inc., Cary, NC, USA).

## Results

### Baseline Demographics

Based on a prospective longitudinal cohort (Figure 1), Figure 2 shows 4 major eGFR profiles which were determined by calculating the average empirical Bayes estimates of all random effects in each patient. We further analyzed the demographic and clinical characteristics of the cohort according to each of the eGFR trajectory patterns (Table 1). Patients with persistent increasing eGFR (T2) were older than patients in the other subgroups, and the T1 group had a higher percentage of smoking and alcohol consumption after *post-hoc* test comparing all pairs of subgroups. Baseline eGFR in the T3 group was lower than that in the other subgroups, and a higher proportion of patients in the late stages of CKD were also found in the T3 group (Table 1).

**Table 1:** Demographic and clinical characteristics of four eGFR patterns.

Parameter^*^	**T0**	**T1**	**T2**	**T3**	***p* value**
**Number of patients**	***n* = 774**	***n* = 1,519**	***n* = 164**	***n* = 1,491**	
**Demographic characteristics**					
Age (years)	76.1 ± 7.0	76.3 ± 7.1	79.9 ± 6.9	75.0 ± 6.9	< 0.001
Gender, male (%)	510 (65.9)	1,155 (76.0)	121 (73.8)	854 (57.3)	< 0.001
Smoking (%)	289 (39.6)	622 (42.5)	57 (36.3)	494 (35.0)	< 0.001
Alcohol (%)	186 (25.5)	446 (30.5)	41 (26.1)	326 (23.1)	< 0.001
Diabetes mellitus (%)	311 (40.2)	552 (36.3)	44 (26.8)	649 (46.0)	< 0.001
Hypertension (%)	538 (69.5)	1,097 (72.2)	108 (65.9)	1,110 (78.7)	< 0.001
Cardiovascular diseases^†^ (%)	103 (13.3)	201 (13.2)	28 (17.1)	159 (11.3)	0.108
Malignancy (%)	80 (11.0)	116 (7.9)	3 (7.3)	123 (8.7)	0.124
**Baseline eGFR, ml/min per 1.73 m**^**2**^ **by MDRD equation**	27.9 ± 9.8	38.5 ± 13.2	58.0 ± 22.3	21.5 ± 14.4	< 0.001
1. ≥ 90	0 (0.0)	5 (0.3)	10 (6.1)	3 (0.2)	< 0.001
2. 60 to < 90	7 (0.9)	84 (5.5)	63 (38.4)	12 (0.8)	
3. 30 to < 60	289 (37.3)	1,092 (71.9)	78 (47.6)	372 (24.9)	
4. 15 to < 30	420 (54.3)	291 (19.2)	11 (6.7)	439 (29.4)	
5. < 15	58 (7.5)	47 (3.1)	2 (1.2)	665 (44.6)	
**Malnourishment indicators**					
Serum albumin < 3.5 g/dL (%)	108 (13.9)	161 (10.6)	36 (21.7)	313 (21.0)	< 0.001
BMI, kg/m^2^	24.6 ± 3.7	24.7 ± 3.5	24.3 ± 4.4	24.5 ± 3.6	0.474
GNRI	105.9 ± 11.1	107.2 ± 10.6	101.4 ± 11.8	103.0 ± 11.4	< 0.001
**Other laboratory data**					
Serum uric acid ≥ 7.2 mg/dL (%)	523 (67.6)	896 (59.0)	97 (59.3)	941 (63.1)	0.013
HbA1c ≥ 6.5% (%)	131 (16.9)	260 (17.1)	7 (4.3)	253 (17.0)	< 0.001
Serum albumin, g/dL	3.9 ± 0.5	4.1 ± 0.5	3.8 ± 0.5	3.8 ± 0.5	< 0.001
**Medications, n (%)**					
ACEIs/ARBs	497 (64.2)	992 (65.3)	116 (70.6)	854 (57.3)	0.001
Insulin	265 (34.2)	380 (25.0)	72 (43.8)	587 (39.4)	< 0.001
OAD	271 (35.0)	501 (33.0)	24 (14.6)	558 (37.4)	< 0.001
Antilipemic agents	172 (22.2)	374 (24.6)	72 (43.9)	362 (24.3)	0.016
Drugs for hypertension	559 (72.2)	1,104 (72.7)	136 (82.9)	1,145 (76.8)	0.024
**Follow-up time, years, median (25th−75th percentile)**	1.8 (1.1–4.5)	2.7 (1.4–4.8)	2.0 (1.3–3.6)	2.1 (1.1–4.0)	< 0.001
**Death (%)**	102 (13.2)	95 (6.3)	7 (4.3)	129 (8.7)	< 0.001

* *Data are presented as mean ± SD or n (%) of participants*.

† *Presence of cardiovascular diseases, i.e., coronary artery disease, stroke, congestive heart failure, arrhythmia, and peripheral arterial disease*.

*T0, gradual eGFR decline; T1, early non-decline and then persistent decline; T2, persistently increasing eGFR; T3, low baseline eGFR and then progressively increasing eGFR; MDRD, Modification of Diet in Renal Disease Study Equation for Estimating Glomerular Filtration Rate; BMI, body mass index; GNRI, Geriatric nutritional risk index; ACEI, Angiotensin-Converting-Enzyme Inhibitor; ARB, Angiotensin Receptor Blocker; OAD, oral antidiabetic*.

Prevalence of nutrition-related risks, including low serum albumin, abnormal BMI, and low GNRI value in both the T2 and T3 groups were higher than those in the T0 and T1 groups. The median follow-up was 2.27 years [interquartile range (IQR), 1.16–4.45]. A total of 333 individuals (8.4%) died at the end of the study (Table 1).

### Adjusted Associations of Trajectory Classes

Because the presence of certain parameters may be correlated with increased or decreased odds of trajectory classes (2), we then examined the association between the eGFR trajectory patterns and confounding factors via the multinomial logistic regression analysis (Table 2). After adjusting confounding factors, compared to the T0 group in the CKD cohort, eGFR trajectory T1 and T2 were associated with higher baseline eGFR values, but eGFR trajectory T3 was less likely to have high baseline eGFR. The estimated odds of being classified into the T1, T2, and T3 groups, rather than the T0 group, were 1.06 (95% CI 1.05–1.07), 1.13 (95% CI 1.11–1.14), and 0.96 (95% CI 0.95–0.97), respectively. Compared to the eGFR trajectory T0 group, the T1 group had higher serum albumin levels, and the T2 group had more elderly people, but the T3 group had a greater likelihood of hypertension. Compared to the T0 group, the eGFR trajectory T2 group had a higher proportion of older patients, while the eGFR trajectory T3 group had a higher proportion of younger patients. More specifically, for each additional year in age, there was a significant increase in the odds of being classified into the T2 group, but there was a decrease in the odds of being classified into the T3 group when compared to the T0 group.

**Table 2:** Multinomial logistic regression to evaluate the association of patients' characteristics with different eGFR trajectory patterns.

**TCVGH_CKD cohort**	**Gradual eGFR decline (T0)**	**Early non-decline and then persistent decline (T1)**		**Persistently increasing eGFR (T2)**		**Low baseline eGFR with the early decline and then progressively increasing eGFR (T3)**	
	**OR**	**OR (95% CI)**	***p* value**	**OR (95% CI)**	***p* value**	**OR (95% CI)**	***p* value**
Age	1.0	1.00 (0.99–1.01)	0.95	1.09 (1.06–1.12)	< 0.001	0.98 (0.97–1.00)	0.01
Male vs. female	1.0	1.27 (1.00–1.62)	0.05	0.90 (0.54–1.50)	0.69	0.91 (0.72–1.15)	0.42
Smoking	1.0	0.85 (0.67–1.09)	0.20	0.69 (0.42–1.14)	0.15	1.00 (0.77–1.29)	0.98
Alcohol	1.0	1.13 (0.88–1.44)	0.33	1.27 (0.75–2.14)	0.38	1.04 (0.81–1.34)	0.73
Diabetes mellitus	1.0	0.93 (0.77–1.13)	0.49	0.79 (0.52–1.22)	0.29	1.12 (0.93–1.36)	0.23
Hypertension	1.0	1.16 (0.94–1.42)	0.16	1.19 (0.78–1.82)	0.43	1.58 (1.28–1.95)	< 0.001
Cardiovascular disease	1.0	1.07 (0.82–1.41)	0.60	1.54 (0.91–2.61)	0.11	0.83 (0.63–1.09)	0.18
Baseline eGFR	1.0	1.06 (1.05–1.07)	< 0.001	1.13 (1.11–1.14)	< 0.001	0.96 (0.95–0.97)	< 0.001
Albumin	1.0	1.38 (1.13–1.69)	0.002	0.93 (0.64–1.33)	0.68	0.84 (0.69–1.02)	0.08
BMI	1.0	0.99 (0.96–1.03)	0.63	1.01 (0.96–1.07)	0.61	1.00 (0.96–1.03)	0.76

*Applied multiple imputation*.

*OR, odds ratio; CI, confidence interval*.

### Clinical Characteristics Based on the Absence or Presence of Malnourishment

During the 12-year study period, 1,872 older adults had complete data with which to identify the status of malnourishment. Among these 1,872 patients, 765 (40.9%) were identified as having malnourishment. The study participants were predominantly male (66.7%), and the mean age was 76.9 years. The most common comorbidities were hypertension (77.7%) and DM (38.8%). Compared with the participants with malnourishment, those without malnourishment were younger, predominantly male, and more likely to have diabetes and/or hypertension. Participants without malnourishment also had higher alcohol consumption, HbA1c, and more prescriptions for hyperlipidemia and hypertension drugs (Table 3).

**Table 3:** Demographic and clinical characteristics between older adults without and with malnourishment.

Parameter^*^	**Overall**	**Without malnourishment**	**With malnourishment**	***p* value**
**Number of patients**	***n* = 1,872**	***n* = 1,107**	***n* = 765**	
**Demographic characteristics**				
Age (years)	76.9 ± 6.7	76.5 ± 6.5	77.6 ± 6.9	0.001
Gender, male (%)	1,249 (66.7)	765 (69.1)	484 (63.3)	0.010
Smoking (%)	706 (37.9)	417 (37.7)	289 (37.8)	0.962
Alcohol (%)	493 (26.5)	318 (28.7)	175 (22.9)	0.006
Diabetes mellitus (%)	727 (38.8)	461 (41.6)	266 (34.8)	0.003
Hypertension (%)	1,454 (77.7)	918 (82.9)	536 (70.1)	< 0.001
Cardiovascular diseases^†^ (%)	224 (12.0)	125 (11.3)	99 (12.9)	0.313
Malignancy (%)	161 (9.1)	97 (8.8)	64 (8.4)	0.977
**Baseline eGFR, ml/min per 1.73 m**^**2**^ **by MDRD equation**	31.1 ± 16.8	31.5 ± 15.4	30.5 ± 18.6	0.199
1. ≥ 90	13 (0.7)	5 (0.5)	8 (1.0)	0.216
2. 60– < 90	82 (4.4)	40 (3.6)	42 (5.5)	
3. 30– < 60	878 (46.9)	563 (50.9)	315 (41.2)	
4. 15– < 30	557 (29.8)	321 (29.0)	236 (30.8)	
5. < 15	342 (18.3)	178 (16.1)	164 (21.4)	
**Malnourishment indicators**				
Serum albumin < 3.5g/dL, (%)	289 (15.4)	46 (4.2)	243 (31.8)	< 0.001
BMI, kg/m^2^	24.1 ± 3.7	26.1 ± 2.9	20.9 ± 2.5	< 0.001
GNRI	104.9 ± 11.2	110.7 ± 7.0	93.5 ± 9.1	< 0.001
**eGFR trajectory (%)**				0.988
T0	349 (18.6)	207 (18.7)	142 (18.6)	
T1	710 (37.9)	461 (41.6)	249 (32.5)	
T2	119 (6.4)	58 (5.2)	61 (8.0)	
T3	694 (37.1)	381 (34.4)	313 (40.9)	
**Other laboratory data**				
Serum uric acid ≥ 7.2 mg/dL (%)	917 (51.9)	644 (58.2)	327 (42.7)	0.331
HbA1c ≥ 6.5%, (%)	398 (21.3)	280 (25.3)	118 (15.4)	< 0.001
Serum albumin, g/dL	3.9 ± 0.6	4.1 ± 0.3	3.5 ± 0.7	< 0.001
**Medications, n (%)**				
ACEIs/ARBs	1,170 (62.5)	704 (63.6)	464 (60.6)	0.303
Insulin	622 (33.2)	340 (30.7)	288 (37.7)	0.105
OAD	667 (35.6)	436 (39.4)	231 (30.2)	< 0.001
Antilipemic agents	472 (25.2)	294 (26.6)	152 (19.9)	0.004
Drugs for hypertension	1,380 (73.7)	808 (73.0)	495 (64.7)	0.005
**Follow-up time, years, median (25th−75th percentile)**	2.7 (1.3–4.3)	2.9 (1.4–4.3)	2.4 (1.2–4.3)	< 0.001
**Death (%)**	139 (7.4)	57 (5.1)	82 (10.7)	< 0.001

* *Data are presented as mean ± SD or n (%) of participants*.

† *Presence of cardiovascular diseases, i.e., coronary artery disease, stroke, congestive heart failure, arrhythmia, and peripheral arterial disease*.

*T0, gradual eGFR decline; T1, early non-decline and then persistent decline; T2, persistently increasing eGFR; T3, low baseline eGFR and then progressively increasing eGFR; MDRD, modification of diet in renal disease study equation for estimating glomerular filtration rate; BMI, body mass index; GNRI, Geriatric nutritional risk index; ACEI, angiotensin-converting-enzyme inhibitor; ARB, angiotensin receptor blocker; OAD, oral antidiabetic*.

### Correlations of eGFR Trajectory and Malnourishment With All-Cause Mortality and Kidney Failure

**Figure 3:**
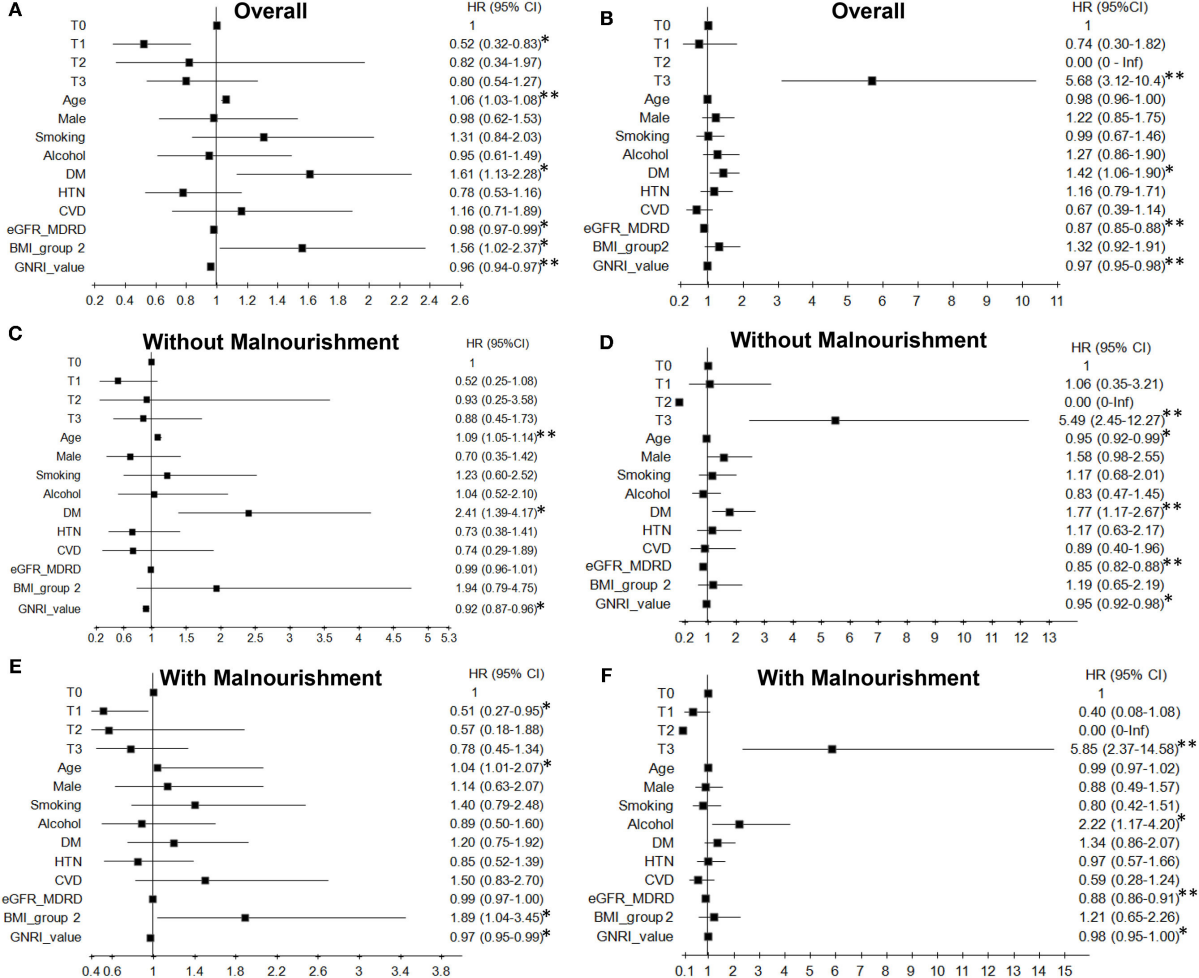
Effects of kidney function trajectories and malnourishment on all-cause mortality **(A,C,E)** and kidney failure **(B,D,F)** among older adults. 1,872 older patients were investigated, and the outcomes were fully adjusted for demographics, laboratory parameters, clinical comorbid conditions, GNRI, and medication. There were no kidney failure events for the eGFR trajectory T2 pattern. HR, Hazard ratio; CI, confidence interval; DM, diabetes mellitus; HTN, hypertension; CVD, cardiovascular diseases; BMI_group 2, BMI cut-off point 24 kg/m^2^. The normal BMI value: male is 19.2–23.7 kg/m^2^ and female is 18.3–22.7 kg/m^2^ (32). *p < 0.05, **p < 0.001.

For all-cause mortality in the overall patients, compared to the T0 group, the eGFR trajectory T1 group had a lower risk (aHR = 0.52, 95% CI 0.32–0.83, *p* = 0.007) after adjusting for all other covariates (Figure 3A). More specifically, there was a lower risk for patients with malnourishment and eGFR trajectory T1 (aHR = 0.51, 95% CI 0.27–0.95, *p* = 0.035) (Figure 3E), but there was no significant risk for mortality in patients with eGFR trajectory T1 and without malnourishment (aHR = 0.52, 95% CI 0.25–1.08, *p* = 0.08) (Figure 3C).

Regarding the risk of kidney failure in the overall patients, eGFR trajectory T3 in patients significantly associated with a higher risk for kidney failure (aHR = 5.68, 95% CI 3.12–10.4, *p* < 0.001) compared to the reference (Figure 3B). In the presence of malnourishment and eGFR trajectory T3, the aHR was 5.85 (95% CI 2.37–14.58; Figure 3F); in patients with eGFR trajectory T3 and without malnourishment, the aHR was 5.49 (95% CI 2.45–12.27; Figure 3D) after compared with the reference.

### Malnourishment and Comorbid Illness Are Risks for All-Cause Mortality and Kidney Failure

Concerning the effect of comorbid illness on all-cause mortality, baseline high eGFR and high GNRI value were significantly associated with a lower risk of death (Figure 3A) and kidney failure (Figure 3B), although there was no GNRI effect on kidney failure in patients with malnourishment (Figure 3F). In the subgroup analysis of eGFR on mortality, high eGFR was not significantly associated with all-cause mortality in the absence (Figure 3C) or presence (Figure 3E) of malnourishment. Patients of older age without or with malnourishment in the CKD cohort were associated with a high risk of death, respectively (without malnourishment: aHR = 1.09, 95% CI 1.05 −1.14, *p* < 0.001; Figure 3C; with malnourishment: aHR = 1.04, 95% CI 1.01–2.07, *p* = 0.011; Figure 3E). However, in the absence of malnourishment, patients with older age had a significantly lower risk of kidney failure than relatively young patients (Figure 3D). Moreover, DM in all patients (Figure 3A) and BMI > 24 in patients with malnourishment were significantly associated with a higher risk of death (Figure 3E). DM in all patients also had a higher risk of kidney failure (Figure 3B). Furthermore, patients with alcohol consumption had a high risk of kidney failure, especially those with malnourishment (Figure 3F). The multiplicative interaction analysis among eGFR trajectory, malnourishment, and comorbidities was significant in all-cause mortality as well as kidney failure. For mortality, patients with DM, eGFR trajectory T1, and BMI > 24 had different associations on the outcome between patients without malnourishment and patients with malnourishment. For kidney failure, patients with older age, alcohol consumption, and DM had different associations on the outcome between patients without and with malnourishment (Supplementary Table 2). The results of formal interaction analysis are almost consistent with those of the aforementioned analysis stratified by malnourishment. Furthermore, this association and predictive value of eGFR trajectory T3 for all-cause mortality were inverse when the initial 3,948 older patients were without selection for malnourishment (Table 4).

**Table 4:** Effects of kidney function trajectories and comorbid illnesses on all-cause mortality among 3,948 older patients with chronic kidney disease without multiple imputation for missing data and without selection for malnourishment.

**Parameters**	**TCVGH_CKD**	
	HR (95% CI)^*^	***p* value**
eGFR trajectory		
T0 (gradual eGFR decline)	1.0 –	–
T1 (early non-decline and then persistent decline)	0.46 (0.35–0.62)	< 0.001
T2 (persistently increasing eGFR)	0.37 (0.17–0.83)	0.02
T3 (low baseline eGFR with early decline and then progressively increasing eGFR)	0.70 (0.54–0.92)	0.009
Age	1.08 (1.06–1.09)	< 0.001
Male	1.16 (0.87–1.56)	0.32
Smoking	1.09 (0.81–1.47)	0.57
Alcohol	0.94 (0.68–1.30)	0.70
DM	1.23 (0.97–1.55)	0.09
HTN	0.56 (0.44–0.72)	< 0.001
CVD	0.99 (0.98–1.39)	0.94
Baseline eGFR	0.99 (0.98–0.99)	0.002
BMI_group 2	1.16 (0.82–1.65)	0.40
GNRI_value	0.97 (0.96–0.98)	< 0.001

* *Fully adjusted for demographics, laboratory parameters, clinical comorbid conditions, GNRI, and medication*.

*HR, hazard ratio; CI, confidence interval; DM, diabetes mellitus; HTN, hypertension; CVD, cardiovascular diseases; BMI_group 2, BMI cut-off point 24 kg/m^2^*.

*The normal BMI value: male is 19.2–23.7 kg/m^2^ and female is 18.3–22.7 kg/m^2^ (32)*.

## Discussion

To the best of our knowledge, this is the first study to characterize the full spectrum of different eGFR trajectory patterns in CKD older adults without or with malnourishment. We also found that longitudinal eGFR change was more useful than baseline eGFR or eGFR decline rate when the observation window was extended. This was tested and estimates for baseline GFR were statistical borderline in the models for both mortality and kidney failure (Figure 3). We characterized four phenotypically distinct functional trajectories. Although eGFR trajectory T0 and T1, respectively, showed a consistently slow decline of eGFR and early non-decline before persistent eGFR decline in the CKD patients (1–3), trajectory T3, which showed low baseline eGFR with an early decline and increasing eGFR in the follow-up period, was notably and highly associated with kidney failure in older adults with malnourishment (Figure 3F). Besides, multimorbidity, abnormal BMI status, and malnutrition were significantly predictive of patients' outcomes in older adults (Figure 3).

The lower association of T1 with mortality is stated to be specifically present among patients with malnourishment. Although the estimate was not statistically significant for patients without malnourishment, it was nearly identical to estimates for the full cohort and the subset with malnourishment. The confounding factor due to malnourishment may specifically present to support the association of T1 with mortality. Those highlighted the intensive and integrated kidney disease care, including low protein diet, control of blood pressure, sugar, hyperlipidemia, correction of metabolic acidosis by sodium bicarbonate, and adequate administration of erythropoietin stimulating agents might slow the renal progression that is independently associated with lower risk for heart failure, myocardial infarction, and peripheral arterial disease (33, 34). Then, circulating the uremic milieu triggering chronic inflammation and oxidative stress-related cardiovascular events and all-cause death might be reduced, especially in the T1 group (35).

Similarly, the HRs for T3 and kidney failure was 5.85 and 5.49 for malnourished and not malnourished older patients, respectively. Both significant differences and with adequate confidence intervals claim that the HR decreased from one group to the other. Those may suggest in the data that these associations meaningfully differ, and again, the determinants might be presented.

Several potential mechanisms may explain the determinant of malnourishment on eGFR trajectory T3-associated kidney failure. First, malnutrition harming age-related multimorbidity, subsequent sarcopenia, and neuroendocrine dysfunction eventually results in an early decreasing and then increasing eGFR, and the vicious cycle also negatively impacts survival and renal outcome (36). Second, in studies conducted by the US Department of Veterans Affairs (VA), 66% of patients died without having composite kidney disease and 34% of patients died after developing kidney disease (2, 10). Most affected patients were older adults. It is plausible that we speculated that the eGFR trajectory patterns and selection of malnourishment would simultaneously involve multimorbidity and nutritional status, and our analysis indeed showed that these factors were correlated with mortality or kidney failure in older adults. Finally, the underlying effect of malnourishment on kidney failure in older people with increasing eGFR in the follow-up period may include undernourishment, sarcopenic obesity (BMI > 24), and multimorbidity rather than underweight status, which can lead to dysregulation or decompensation of renal function (4). This finding is also compatible with low creatinine appearance in kidney disease cachexia in the criteria of low muscle mass, especially in older adults (37).

Initial low eGFR has been attributed to several concordant and discordant comorbid conditions, which had direct or indirect associations with the outcome (2). Baseline malnourishment status may play a prominent role in the mechanisms underlying the effect of increasing eGFR trajectory patterns on kidney failure before the death event in older people. We used baseline eGFR and eGFR trajectory patterns to test the outcomes and found high or low eGFR value presented a considerable clinical challenge, which may frustrate attempts to achieve optimal outcomes in patients with coexisting diseases.

A major strength of this study was the differences in mortality rates and kidney failure among four eGFR trajectory patterns which have not been previously done in the literature. Secondly, we showed that the most striking finding in the present study was the association of eGFR trajectory and comorbid conditions with the risk of outcomes which is modified by malnourishment status. It is not inferior to the diagnostic performance of several available tests, such as integrated discrimination index (IDI) or net reclassification improvement (27, 38) for the candidate biomarkers (longitudinal measurements of cystatin C) in terms of accuracy of risk prediction (6). Thirdly, our method of analysis was capable of fully interpreting the nonlinear eGFR trajectories or a prolonged period of non-progression in intrinsic or extrinsic renal diseases.

There were some potential limitations in the present study that should be addressed. Firstly, there were not enough variables for comparison due to the large size of the cohort. Other possible confounding factors included several concordant and discordant comorbid conditions. Since albuminuria and proteinuria were not included in our dataset, the accuracy of nutritional indicators such as albumin and GNRI is questionable. Especially in patients with nephrotic proteinuria, these nutritional indicators may not adequately reflect nutritional status. Secondly, this study included mostly Taiwanese individuals with a highly selected population, and thus the results may not be generalizable to less narrowly defined populations. Thirdly, old age seemed to have protective effects on kidney failure in the CKD cohort, and this finding was consistent with previous findings. The age distribution was the same between the results obtained using multiple imputations and those without any imputations (Supplementary Figure 2). Fourth, in clinical practice, it is necessary to early diagnose the risk of nutritional disorders and kidney failure for starting treatment promptly and preventing their progression. However, it takes years to diagnose which of the four eGFR trajectory patterns a patient falls into. Fifth, the initiation, dose increase, and discontinuation of drugs that affect glomerular filtration such as angiotensin-converting-enzyme inhibitor and angiotensin receptor blocker may influence the eGFR trajectory pattern. However, it is difficult to present in this study.

In conclusion, eGFR trajectories were shown to be a valuable prognostic indicator for predicting outcomes in older adults with CKD. An integrated kidney disease care program could have a notable beneficial effect on patients' mortality and kidney failure, based on a comparison with gradual eGFR decline. Increasing eGFR trajectory in the later period was shown to be a high-risk factor for kidney failure in older CKD patients. These phenomena may be due to multimorbidity, abnormal BMI status, and malnutrition.

## Data Availability Statement

The original contributions presented in the study are included in the article/Supplementary Material, further inquiries can be directed to the corresponding authors.

## Ethics Statement

The studies involving human participants were reviewed and approved by Ethics Committee of Taichung Veterans General Hospital (Nos. CF13015, CF13015-1, CF13015-2, CF13015-3, and CE12252-1). The patients/participants provided their written informed consent to participate in this study.

## Author Contributions

S-CW wrote the manuscript. C-MC, Y-CC, M-JW, and D-CT conceived and designed the experiments and contributed to the discussion and manuscript revision. C-MC performed the analyses. S-CW, M-JW, and D-CT performed the experiments and collected the data. M-JW and D-CT conceived the study and are the guarantors of this publication. All authors reviewed the manuscript, contributed to the article, and approved the submitted version.

## Funding

We are also deeply indebted to Taichung Veterans General Hospital, Taichung for providing the grants for this study (TCVGH-YM1050101, TCVGH-1068201B, TCVGH-YM1060103, TCVGH-1078201B, TCVGH-YM1070101, TCVGH-1088201B, TCVGH-YM1080103, TCVGH-1098201B, TCVGH-1108201B, TCVGH-1108202D, and TCVGH-YM1090105). This study was also supported by Taiwan's Ministry of Science and Technology (MOST 106-2314-B-075A-003) and the Center for Intelligent Drug Systems and Smart Bio-devices (IDS2B) from the Featured Areas Research Center Program within the framework of the Higher Education Sprout Project by the Ministry of Education (MOE) in Taiwan and the Foundation for Poison Control.

## Conflict of Interest

The authors declare that the research was conducted in the absence of any commercial or financial relationships that could be construed as a potential conflict of interest.

## Publisher's Note

All claims expressed in this article are solely those of the authors and do not necessarily represent those of their affiliated organizations, or those of the publisher, the editors and the reviewers. Any product that may be evaluated in this article, or claim that may be made by its manufacturer, is not guaranteed or endorsed by the publisher.
