# Supplementary material for: Trajectory of Estimated Glomerular Filtration Rate and Malnourishment Predict Mortality and Kidney Failure in Older Adults With Chronic Kidney Disease
Source: Front Med (Lausanne). 2021 Nov 29;8:760391. doi: 10.3389/fmed.2021.760391 (PMC8666586; doi:10.3389/fmed.2021.760391)
Supplement: Supplementary file 1 [file Data_Sheet_1.pdf]

Trajectory of Estimated Glomerular Filtration Rate and Malnourishment Predict Mortality and Kidney Failure in Older Adults With Chronic Kidney Disease

Shuo-Chun Weng[1,2,3], Chyong-Mei Chen[4], Yu-Chi Chen[5], Ming-Ju Wu[1,2,6,7,8], Der-Cherng Tarng[3,9,10,11*]

Supplementary Figure 1. Polygonal lines are the subject-specific longitudinal trajectories for eGFR_MDRD of 35 randomly selected subjects in each group. Each smooth curve is the average of the observed trajectories in the group corresponding to the same color.

Supplementary Table 1. Patients and levels of indicators for malnourishment in 3,948 participants.

Supplementary Table 2. Interaction analyses of risk for overall mortality and kidney failure in older adults without or with malnourishment.

Supplementary Figure 2. Histogram of age distribution before and after imputation.

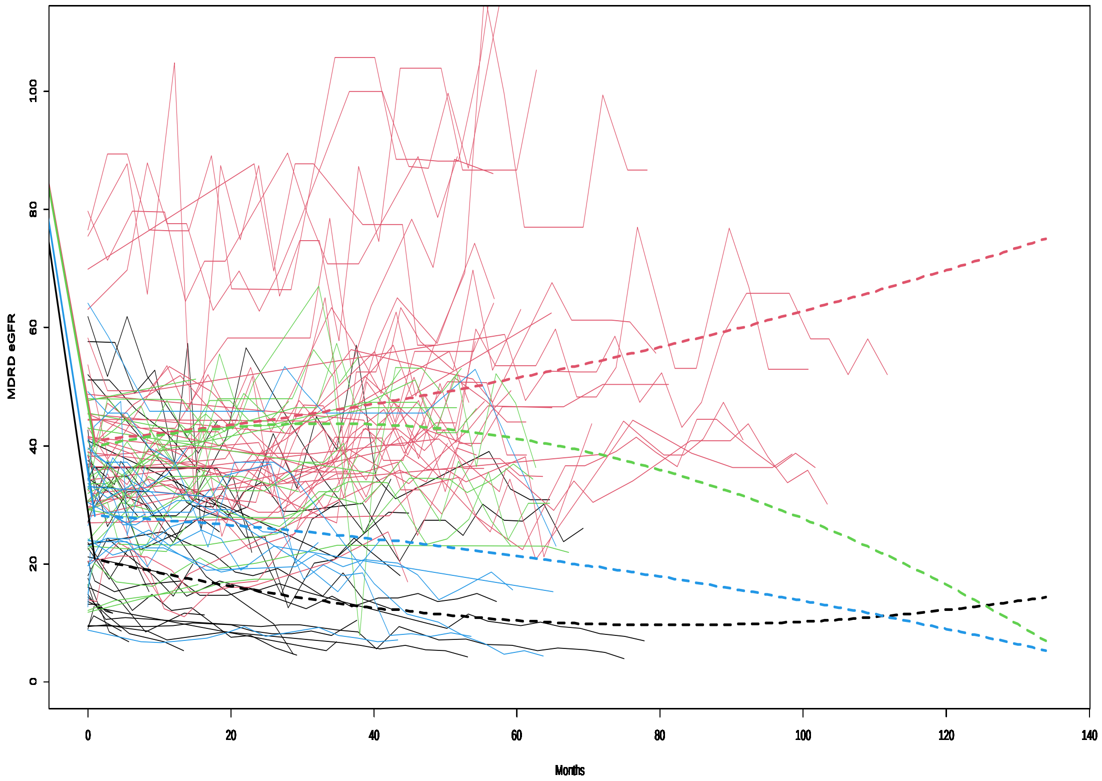

Supplementary Figure 1. Polygonal lines: the subject-specific longitudinal trajectories for eGFR_MDRD of 35 randomly selected subjects in each group. Smooth curve: the average of the observed trajectories in the group corresponding to the same color. Blue curves: gradual decline (T0), Green curves: early non-decline and then persistent decline (T1), Red curves: persistent increase (T2), and Black curves: low baseline eGFR and then progressive increase (T3). The dashed curves represent the estimated group-specific trajectories.

Discussion:

To catch the trajectories of all patients, the commonly used simple linear regression for the eGFR in time *t* (i.e., a straight line) is not good enough to describe the individual variation (heterogeneity). Hence we are motivated to apply the linear mixed-effects model. Furthermore, the preliminary figure of eGFR versus examination time showed that some patients' eGFR decreased oscillatory and then increased, but some increased gradually. Hence, we consider the linear mixed-effects model with a polynomial of degree 2, that is, a parabola with the following model

$$Y_t = \beta_0 + b_0 + \beta_1 t + b_1 t + \beta_2 t^2 + b_2 t^2 + \varepsilon_t,$$

where Y_t is the eGFR of a patient measured at time *t* since recruitment, ε_t is an error term following $\mathrm{N}(0, \sigma^2)$, β_0, β_1 and β_2 are fixed effects, and b_0, b_1 and b_2 are random effects following a trivariate normal distribution with mean zero and variance-covariance matrix Σ, a positive definite 3 by 3 symmetry matrix. Here,

$$\Sigma = \begin{bmatrix} \sigma_0^2 & \sigma_{01} & \sigma_{02} \\ \sigma_{01} & \sigma_1^2 & \sigma_{12} \\ \sigma_{02} & \sigma_{12} & \sigma_2^2 \end{bmatrix},$$

$\sigma_k^2 = \mathrm{var}(b_k), k = 0, 1, 2$ and σ_{kj}^2 are the covariance of b_k and $b_j, k \neq j$. The sample size is large enough to analyze our considered model (Diggle, Liang and Zeger,

1994; Laird and Ware, 1982). After obtaining the empirical Bayes estimates of each patient's random effects, each individual's pattern of eGFR in time could be estimated. By utilizing the properties of a parabola, we classify all patients into 4 groups gradual decline (T0), early non-decline and then persistent decline (T1), persistent increase (T2), and low baseline and then progressive increase (T3).

Supplementary Figure 1 shown in the following presents the plot of eGFR measurements for 35 randomly selected patients in each group. Visualization of these curves indicates that the proposed 4-group classification could adequately describe the trajectories of the patients. This figure also indicates that the commonly used linear pattern for the eGFR in time *t* is not good enough.

References:

Diggle, P. J., Liang, K.-Y. and Zeger, S. L. (1994). *Analysis of Longitudinal Data*, Oxford University Press, New York.

Laird, N. M. and Ware, J. H. Random-effects models for longitudinal data, *Biometrics.* (1982) **38**: 963–974.

Although most follow-up times were less than 60 months, there were still many were over 60 months. For example, in the T3 group, 429 patients were followed over 60 months, which could provide sufficient information for inferring the pattern of longitudinal data on the observation period. Consequently, individuals in T3 were predicted to have a low and steady lowering of eGFR but the line for some reason makes an up-turn at 80 months or so.

Supplementary Table 1. Patients and levels of indicators for malnourishment in 3,948 participants.

	GNRI<98	Albumin <3.0 g/dL	BMI <22 kg/m^2
No. of nonmissing	1,665	1,706	1,831
One indicator only	389	140	574
Two indicators			
GNRI<98	-	98	240
Albumin <3.0 g/dL	98	-	34
BMI <22 kg/m^2	240	34	-
Three indicators	34	34	34

GNRI, Geriatric Nutritional Risk Index; BMI, body mass index

Supplementary Table 2. (A) Interaction analyses of risk for overall mortality in older adults without or with malnourishment*

	**Overall**		**Without Malnourishment**		**With Malnourishment**	
**Parameters**	**HR (95% CI)**	***p* value**	**HR (95% CI)**	***p* value**	**HR (95% CI)**	***p* value**
eGFR trajectory						
T0 (Gradual eGFR decline)	1.00 -	-	1.00 -	-	1.00 -	-
T1 (Early non-decline and then persistent decline)	0.52 (0.32–0.84)	0.008	0.52 (0.25–1.08)	0.08	0.51 (0.27–0.95)	0.035
T2 (Persistently increasing eGFR)	0.81 (0.34–1.97)	0.65	0.93 (0.25–3.58)	0.92	0.57 (0.18–1.88)	0.36
T3 (Low baseline eGFR with early decline and then progressively increasing eGFR)	0.83 (0.55–1.27)	0.40	0.88 (0.45–1.73)	0.70	0.78 (0.45–1.34)	0.36
Age	1.06 (1.03–1.08)	<0.001	1.09 (1.05–1.14)	< 0.001	1.04 (1.01–2.07)	0.011
Male	0.98 (0.63–1.54)	0.94	0.70 (0.34–1.42)	0.33	1.14 (0.63–2.07)	0.66
Smoking	1.30 (0.84–2.01)	0.25	1.23 (0.60–2.52)	0.57	1.40 (0.79–2.48)	0.25
Alcohol	0.96 (0.62–1.50)	0.86	1.04 (0.52–2.10)	0.92	0.89 (0.50–1.60)	0.7
DM	1.61 (1.14–2.29)	0.007	2.41 (1.39–4.17)	0.002	1.20 (0.75–1.92)	0.46
HTN	0.79 (0.53–1.16)	0.23	0.73 (0.38–1.41)	0.35	0.85 (0.52–1.39)	0.52
CVD	1.15 (0.70–1.87)	0.58	0.74 (0.29–1.89)	0.54	1.50 (0.83–2.70)	0.18
Baseline eGFR	0.98 (0.97–1.00)	0.019	0.99 (0.96–1.01)	0.21	0.99 (0.97–1.00)	0.11
BMI_group 2	1.74 (1.06–2.85)	0.027	1.94 (0.79–4.75)	0.15	1.89 (1.04–3.45)	0.038
GNRI_value	0.96 (0.94–0.98)	< 0.001	0.92 (0.87–0.96)	<0.001	0.97 (0.95– 0.99)	0.01
Malnourishment	1.31 (0.71–2.44)	0.39				

*Fully adjusted for demographics, laboratory parameters, clinical comorbid conditions, GNRI, and medication (Cause-specific Cox model).
HR, Hazard ratio; CI, confidence interval; DM, diabetes mellitus; HTN, hypertension; CVD, cardiovascular diseases; BMI_group 2, BMI cut-off point 24 kg/m^2; The normal BMI value: male is 19.2–23.7 kg/m^2 and female is 18.3–22.7 kg/m^2 (Department of Health, Executive Yuan, Taiwan, R.O.C.). [Spindle 2009 health education advocacy plan survey summary report - The definition of body mass index in adults in Taiwan. February 2009; 36(1): 23–26].

Supplementary Table 2. (B) Interaction analyses of risk for kidney failure in older adults without or with malnourishment[†]

	**Overall**		**Without Malnourishment**		**With Malnourishment**	
**Parameters**	**HR (95% CI)**	***p* value**	**HR (95% CI)**	***p* value**	**HR (95% CI)**	***p* value**
eGFR trajectory						
T0 (Gradual eGFR decline)	1.00 -	-	1.00 -	-	1.00 -	-
T1 (Early non-decline and then persistent decline)	0.74 (0.30–1.82)	0.51	1.06 (0.35–3.21)	0.92	0.40 (0.08– 1.08)	0.28
T2 (Persistently increasing eGFR)	0 (0–Inf)	0.98	0 (0–Inf)	0.98	0 (0–Inf)	0.98
T3 (Low baseline eGFR with early decline and then progressively increasing eGFR)	5.68 (3.11–10.34)	<0.001	5.49 (2.45–12.27)	<0.001	5.85 (2.37–14.58)	<0.001
Age	0.98 (0.96–1.00)	0.06	0.95 (0.92–0.99)	0.009	0.99 (0.97–1.02)	0.70
Male	1.22 (0.85–1.75)	0.29	1.58 (0.98–2.55)	0.06	0.88 (0.49–1.57)	0.66
Smoking	1.00 (0.67–1.49)	0.996	1.17 (0.68–2.01)	0.58	0.80 (0.42–1.51)	0.49
Alcohol	1.26 (0.87–1.88)	0.26	0.83 (0.47–1.45)	0.50	2.22 (1.17–4.20)	0.014
DM	1.43 (1.07–1.91)	0.017	1.77 (1.17–2.67)	0.006	1.34 (0.86–2.07)	0.19
HTN	1.16 (0.78–1.71)	0.47	1.17 (0.63–2.17)	0.61	0.97 (0.57–1.66)	0.91
CVD	0.67 (0.39–1.14)	0.14	0.89 (0.40–1.96)	0.77	0.59 (0.28–1.24)	0.17
Baseline eGFR	0.87 (0.85–0.88)	<0.001	0.85 (0.82–0.88)	<0.001	0.88 (0.86–0.91)	<0.001
BMI_group 2	1.26 (0.83–1.92)	0.27	1.19 (0.65–2.19)	0.57	1.21 (0.65–2.26)	0.55
GNRI_value	0.96 (0.94–0.98)	<0.001	0.95 (0.92–0.98)	0.002	0.98 (0.95–1.00)	0.05
Malnourishment	0.90 (0.56–1.44)	0.65				

[†]Fully adjusted for demographics, laboratory parameters, clinical comorbid conditions, GNRI, and medication (Cause-specific Cox model).

HR, Hazard ratio; CI, confidence interval; DM, diabetes mellitus; HTN, hypertension; CVD, cardiovascular diseases; BMI_group 2, BMI cut-off point 24 kg/m^2; The normal BMI value: male is 19.2–23.7 kg/m^2 and female is 18.3–22.7 kg/m^2 (Department of Health, Executive Yuan, Taiwan, R.O.C.). [Spindle 2009 health education advocacy plan survey summary report - The definition of body mass index in adults in Taiwan. February 2009; 36(1): 23–26]. There were no kidney failure events for the eGFR trajectory T2 pattern.

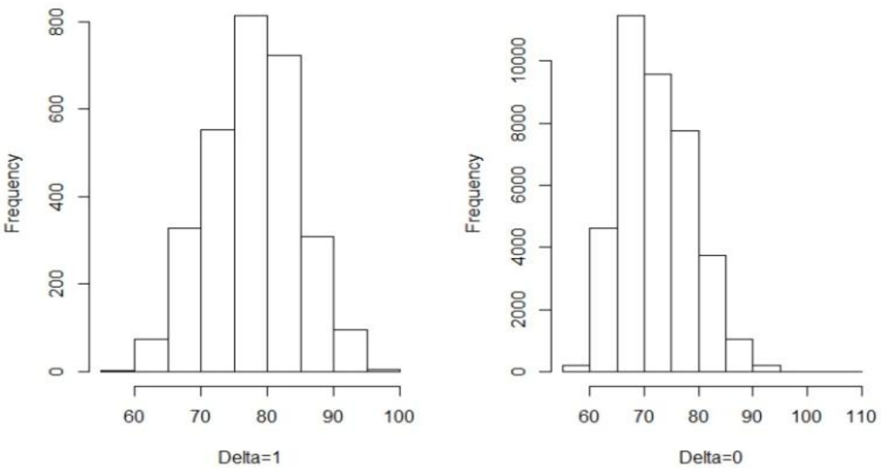

A. Histogram of age distribution before imputation

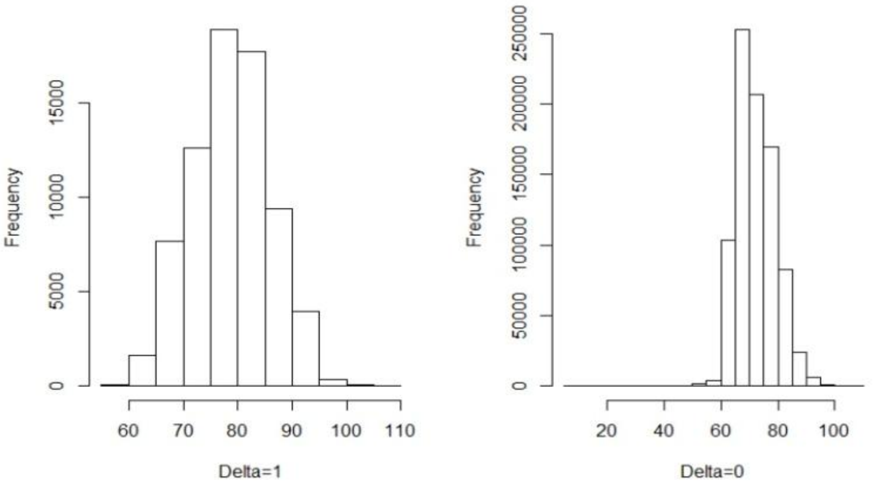

B. Histogram of age distribution after imputation

Supplementary Figure 2. Histogram of age distribution before and after imputation.
